# Brown Adipose Stem Cell-Loaded Resilin Elastic Hydrogel Rebuilds Cardiac Function after Myocardial Infarction via Collagen I/III Reorganisation

**DOI:** 10.3390/gels10090568

**Published:** 2024-08-31

**Authors:** Le Zhao, Huaying Liu, Rui Gao, Kaihui Zhang, Yuxuan Gong, Yaya Cui, Shen Ke, Jing Wang, Haibin Wang

**Affiliations:** 1 College of Life Sciences and Bioengineering, School of Physical Science and Engineering, Beijing Jiaotong University, Beijing 100044, China23121758@bjtu.edu.cn (Y.C.);; 2 Department of Wound Infection and Drug, Army Medical Center of PLA (Daping Hospital), Army Medical University, Chongqing 400042, China; 3 School of Life Sciences, Inner Mongolia University, Hohhot 010000, China; 4 Institute of Health Service and Transfusion Medicine, Academy of Military Medical Sciences, Beijing 100850, China; 5 State Key Laboratory of Toxicology and Medical Countermeasures, Beijing Institute of Pharmacology and Toxicology, Beijing 100850, China

**Keywords:** resilin, injectable hydrogel, myocardial tissue engineering, brown adipose-derived stem cells, myocardial infarction

## Abstract

Irreversible fibrosis following myocardial infarction (MI) stiffens the infarcted myocardium, which remains challenging to restore. This study aimed to investigate whether the injectable RLP12 hydrogel, derived from recombinant resilin protein, could serve as a vehicle for stem cells to enhance the function of the infarcted myocardium. The RLP12 hydrogel was prepared and injected into the myocardium of rats with MI, and brown adipose-derived mesenchymal stem cells (BADSCs) were loaded. The survival and differentiation of BADSCs in vivo were investigated using immunofluorescence one week and four weeks after treatment, respectively. The heart function, MI area, collagen deposition, and microvessel density were further assessed four weeks after treatment through echocardiography, histology, immunohistochemistry, and immunofluorescence. The RLP12 hydrogel was prepared with a shear modulus of 10–15 kPa. Four weeks after transplantation, the RLP12 hydrogel significantly improved cardiac function by increasing microvessel density and reducing infarct area size and collagen deposition in MI rats. Furthermore, the distribution ratio of collagen III to I increased in both the centre and edge areas of the MI, indicating the improved compliance of the infarct heart. Moreover, the RLP12 hydrogel also promoted the survival and differentiation of BADSCs into cardiac troponin T- and α-smooth muscle-positive cells. The RLP12 hydrogel can be utilised as an injectable vehicle of BADSCs for treating MI and regulating collagen I and III expression profiles to improve the mechanical microenvironment of the infarct site, thereby restoring heart function. The study provides novel insights into the mechanical interactions between the hydrogel and the infarct microenvironment.

## 1. Introduction

The mortality rate of cardiovascular diseases remains the highest [1]. Myocardial infarction (MI) is a disease with a poor prognosis that inevitably leads to heart failure. In the later stage of MI, collagen I stacks at the infarct tissue, the infarcted myocardial tissue develops fibrosis, and the compensatory hypertrophy of the residual myocardium causes the loss of contractility, irreversible ventricular remodelling, and heart failure [2]. The restricted regenerative capacity of the heart cannot restore or replace damaged tissue, causing local tissue death and ultimately culminating in mortality [3]. Various therapeutics, such as drug thrombolysis and interventional approaches, have been used in clinical scenarios, with varying degrees of success, concentrating on restoring blood perfusion; only a few methods have been proposed to improve the local microenvironment of MI [4,5].

Cell transplantation has shown great potential in enhancing heart function [6]. Several stem cell types, such as mesenchymal, embryonic, adipose mesenchymal, and induced pluripotent stem cells have been used to treat MI through myocardial regeneration [7,8].

Brown adipose-derived mesenchymal stem cells (BADSCs) have advantages including broad availability, low immunogenicity, notable myocardial differentiation capacity, and paracrine effects and are available for the cardiac tissue engineering of seed cells to restore damaged myocardium [9,10]. The infarct site is harsh and lacks a supportive matrix for cells. By delivering cells alone, it is difficult to achieve long-term cell retention and sufficient cell differentiation; therefore, other approaches are required.

Injectable cardiac tissue engineering is an appealing method to improve the harsh microenvironment of the infarcted myocardium, which can be regulated at the molecular, cellular, and individual levels and can directly deliver drugs to the MI site [11,12,13]. Applying carriers to load cells and cytokines can achieve proangiogenic and anti-fibrotic effects. Among the previously proposed carriers, hydrogels have the unique capacity to construct a microenvironment that can resist rigorous damage and avoid the degradation or rapid inhibition of loaded substances [14]. Vectors are crucial for protection, delivering loads, and improving therapeutic efficacy.

A hydrogel is one of the hot spots in the research field of biomaterials and regenerative medicine, and research progresses rapidly. Recently, scientists investigated the thermal performance and mechanical stability of methacrylic acid porous hydrogels in an aqueous medium at different initial temperatures and hydrogel volume fraction using the molecular dynamics simulation [15]. In addition, hydrogels have been developed as a promising vector carrier. Some injectable hydrogels are administered in a liquid state to the infarcted region and then undergo a rapid change from liquid to gel state owing to the unique microenvironment conditions. Materials such as collagen, dECM, polyethylene glycol (PEG), Polyvinyl Alcohol (PVA), and chitosan are used in injectable treatments [16]. Brown adipose stem cells that have been loaded in a pH-sensitive chitosan hydrogel used for myocardial regeneration showed a high cell survival rate [17]. In addition, alginate gels, specifically Algisyl LVRTM [18] and IK-5001 [19], and decellularized matrix hydrogels, known as VentriGel [20], have been approved and have progressed into clinical trials, demonstrating notable therapeutic effects. Studies have identified their beneficial properties, such as rich sources and low immunogenicity. However, reports on the mechanical variation of biomaterials used for treating MI are limited.

Myocardial tissue fibrosis is accompanied by extracellular matrix (ECM) replacement with denatured collagen. These detrimental matrix changes lead to poor mechanical properties and a less conducive environment for recovery (systolic dysfunction and arrhythmia) [21]. The mechanical clues of fibrotic microenvironment are essential to cell–ECM and cell–cell reactions [22]. Elastomeric hydrogels with favorable elasticity and extensibility can improve the MI microenvironment by providing mechanical support and replacing the ECM. Its intervention can improve local myocardial movement disorders in the infarcted tissue, alleviate stress on the ventricular wall, and facilitate damage repair [23,24]. A correlation between the mechanical properties of hydrogels and the stress of the infarcted ventricular wall has been identified, and a stiffness of less than 50 kPa can reduce muscle fibre stress [24]. The synthetic elastic hydrogel poly (octamethylene maleate (anhydride) citrate) (POMAC) was confirmed to achieve provascularisation in rat and swine models of MI [25]. The research on mechanical clues is essential for the growth of cardiomyocytes. When the substrates with a range of elastic moduli reach a similar stiffness to native tissue, the behaviour and contractile function is promoted [26].

Resilin, an excellent natural elastin, has been further developed. Natural resilin, a water-soluble elastic protein found in insect arthropods, consists of a structurally flexible and flowable array of randomly oriented polypeptide chains [27], which are interconnected through the covalent crosslinking of dityrosine and trityrosine to form a stable structure [28]. It has exceptional mechanical properties, including a low modulus of elasticity, a high-tensile strain capacity, and outstanding durability [29]. The tensile modulus of natural resilin is 600–2000 kPa [30], and its resilience can reach 92% [31]. The protein structure includes three exons: N-terminal elastic, chitin-binding, and C-terminal elastic domains [32]. Both exons 1 and 3 contain a large number of repetitive sequences that provide resilin elasticity. Thus, exon 1 may be a critical structural domain for providing resilience [33,34].

The resilin-like protein (RLP) has strong mechanical and biological functions obtained by repeating the core elastic sequence of resilin exon 1 (GGRPSDSYGAPGGGN) 12 times. RLP-based hydrogels obtained using trimethylolpropane (THP) as a crosslinking agent demonstrate remarkable resilience and facilitate the attachment and growth of human mesenchymal stem cells [35] while being associated with good biocompatibility [36]. RLP-derived biomaterials have been effectively applied in the fields of tissue engineering and drug delivery, particularly in tissue engineering research involving blood vessels [37], vocal cords [38], cartilage [39], and muscles [40].

**Figure 1:**
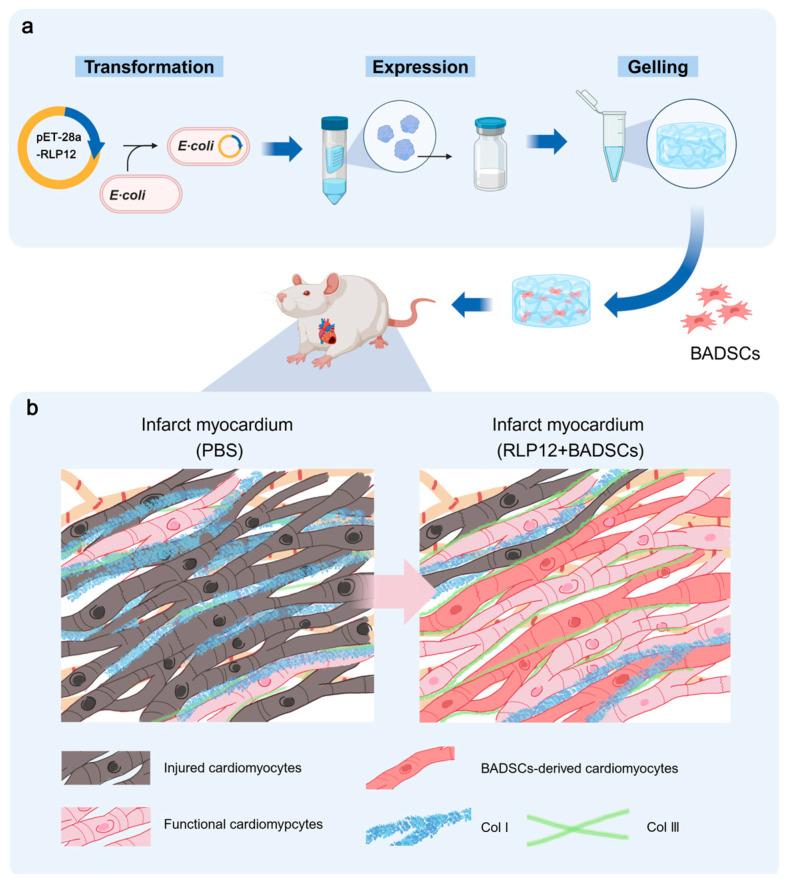
A schematic demonstrating the preparation and application of the RLP12-based delivery system. (**a**) Schematic image for experimental design. (**b**) Illustrations of infarcted myocardium after treatment.

In this study, an injectable RLP12 hydrogel with mechanical properties is synthesised, and whether the hydrogel could serve as a vehicle for stem cells to enhance the function of the infarcted myocardium is investigated. The study is designed as shown in Figure 1.

## 2. Results and Discussion

### 2.1. Synthesis of RLP12 Expression Vector and Protein Expression

**Figure 2:**
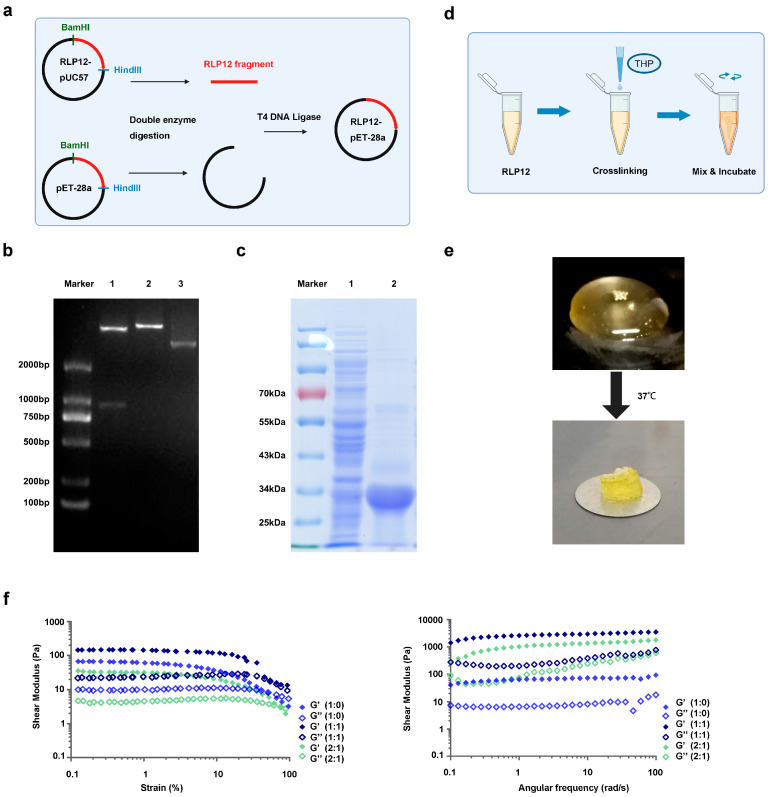
The preparation and characterisation of the RLP12 hydrogel. (**a**) A schematic of the construction of the RLP12 expression vector. (**b**) The identification of the pET-28a-RLP12 recombinant clone vector by dual enzyme digestion. (Lane M: DNA marker; Lane 1: pET-28a-RLP12 plasmid digested by BamH I and Hind III; Lane 2: pET-28a-RLP12 plasmid digested by Hind III; Lane 3: pET-28a-RLP12 plasmid). (**c**) The purification of the RLP12 protein using SDS-PAGE. (Lane M: Prestained protein marker; Lane 1: cell supernatant of bacterial disruption; Lane 2: eluate of target protein). (**d**) A schematic of the process of RLP12 solution gelation. (**e**) RLP 12 solution gelation. (**f**) The oscillatory shear rheological characterisation of resilin hydrogel materials based on RLP12. (Left: strain sweep measurements; right: frequency sweep measurements).

A schematic of the construction of the RLP12 expression vector is shown in Figure 2a. The RLP12 gene sequence was synthesised using the pUC57 plasmid as a vector. After transformation with the recombinant vector pET28a-RLP12, transformants were selected and identified. The results showed that the small fragment band of lane 1 was approximately 800 bp, which is consistent with RLP12, indicating that the expression strain *E. coli* BL21 (DE3)-pET-28a-RLP12 was successfully constructed (Figure 2b). The RLP12 protein eluate collected after Ni^+^-column purification was subjected to sodium dodecyl sulphate-polyacrylamide gel electrophoresis (SDS-PAGE) (Figure 2c). There is a clear protein band between 25 and 34 kDa, which is that of the recombinant RLP12 protein.

### 2.2. Preparation and Mechanical Properties Identification of RLP12 Hydrogel

Three kinds of RLP12 hydrogels were prepared according to the ratios of RLP12 to PEG: RLP12, PEG = 1:0; RLP12, PEG = 1:1; and RLP12, PEG = 2:1 (Figure 2d). After incubation at 37 °C for 15 min, all the solutions formed the light-yellow translucent gels and exhibited viscoelasticity. Figure 2e shows RLP12, PEG = 1:0 as an example. Tests involving stress, frequency, and time scanning were conducted to investigate the in situ oscillatory shear rheological mechanics of samples. Over time, the energy storage modulus of the three samples continued to increase and gradually and steadily reached approximately 10–15 kPa. The stability of the hydrogel was further confirmed using a frequency-scanning experiment. Over the entire frequency range of the experiment (0.1–100 rad/s), the elasticity was higher than the viscosity, and the storage modulus (G’) was always higher than the loss modulus (G’’). This indicates that the three RLP12 hydrogel groups formed by crosslinking exhibited elastic solid behaviour (Figure 2f).

### 2.3. Cardiac Function

**Figure 3:**
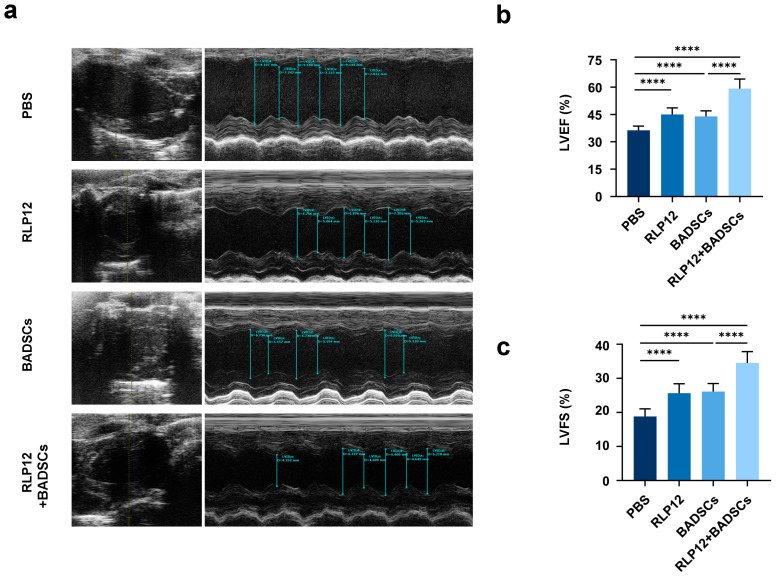
The evaluation of the cardiac function of rats with MI using cardiac ultrasound at 4 weeks after injection. (**a**) Representative echocardiographic images of rats from the different treatment groups. (**b**) LVEF; **** *p* < 0.0001. (**c**) LVFS. **** *p* < 0.0001.

An ultrasound cardiogram was used to assess the heart function of rats with MI four weeks after treatment (Figure 3a). The left ventricular ejection fraction (LVEF) in the PBS-treated animals was 36.3 ± 2.3%, in the RLP12 hydrogel-treated animals was 45.1 ± 3.6%, in the BADSCs-treated animals was 43.9 ± 3.1%, and in the RLP12 hydrogel + BADSCs-treated animals was 59.2 ± 5.2%. Statistical analyses revealed that compared with those in the PBS group, the LVEF levels of the other three groups were significantly increased. Furthermore, the LVEF levels associated with the RLP12 hydrogel + BADSC group were considerably higher than those of the other three groups (Figure 3b).

Our results showed that compared with the PBS-administered group, the left ventricular short-axis shortening index (LVFS) was significantly increased in the other three groups. The LVFS values were as follows: 18.8 ± 2.2% in the PBS; 25.6 ± 2.8% in the RLP12 hydrogel; 26.1 ± 2.4% in the BADSC; and 34.5 ± 3.3% in the RLP12 hydrogel + BADSC groups. The LVFS in the RLP12 hydrogel + BADSC group was significantly higher than that in the other groups (Figure 3c). Thus, delivering RLP hydrogels, BADSCs, or RLP12 hydrogel + BADSCs to the infarcted myocardium of rats can substantially enhance cardiac function. The RLP12 hydrogel showed promising characteristics as a BADSC carrier for intramyocardial injection. Compared with BADSCs or RLP hydrogel alone, RLP12 hydrogel + BADSCs had the most pronounced effect in improving cardiac function.

### 2.4. MI Area Size and Fibrosis Level

**Figure 4:**
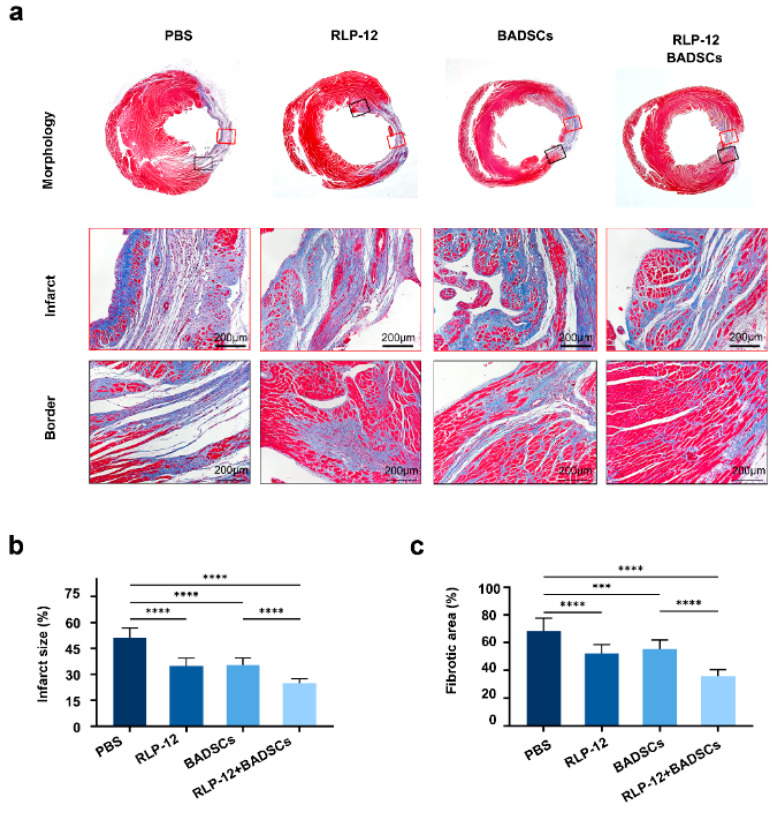
Fibrosis levels assessed using Masson’s trichrome staining. (**a**) The Masson’s trichrome staining of heart slices from a rat with myocardial infarction at four weeks of treatment (scale bar = 200 µm). (**b**) The statistical analysis of the myocardial infarction area, **** *p* < 0.0001. (**c**) The statistical analysis of the collagen deposition level, *** *p* < 0.001, **** *p* < 0.0001.

After four weeks of treatment, cardiac tissue sections were sampled and subjected to Masson’s trichrome staining (Figure 4a). The MI area was quantified by measuring the ratio of the endocardial length in the infarcted area to the left ventricular endocardial circumference. The results showed that the infarct area was 51.1 ± 5.6% in the PBS, 34.9 ± 4.4% in the RLP12 hydrogel, 35.4 ± 4.0% in the BADSCs, and 24.8 ± 2.5% in the RLP12 hydrogel + BADSC groups (Figure 4b). There was a significant reduction in the MI area of all experimental groups compared to that in the PBS group at 4 weeks. Furthermore, the MI area of the RLP12 hydrogel + BADSC group was significantly reduced compared to that of the other three groups, suggesting that injecting the RLP12 hydrogel with BADSCs was the most effective approach. Alternatively, the level of collagen deposition in the PBS group was 68.3 ± 9.5%, in the RLP12 hydrogel group was 52.4 ± 6.2%, in the BADSC group was 55.6 ± 6.4%, and in the RLP12 hydrogel + BADSC group was 35.9 ± 4.8% (Figure 4c). The collagen deposition level of the RLP12 hydrogel + BADSC group was lower than that of the RLP12 hydrogel and BADSC groups, indicating that delivering BADSCs with the RLP12 hydrogel was the most effective in alleviating the degree of fibrosis in rats with MI.

### 2.5. Distribution Levels of Type I Collagen in the Infarct Area and Border Area

**Figure 5:**
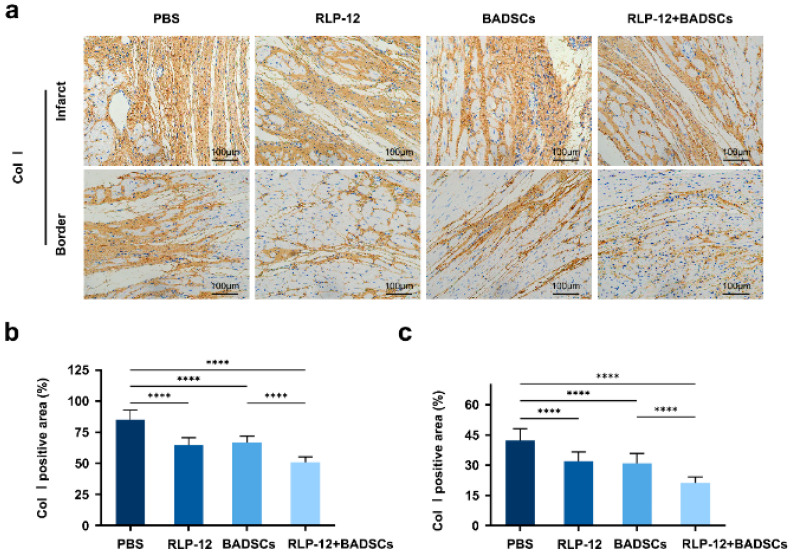
The distribution level of type I collagen in the myocardial infarction and border zone at 4 weeks after treatment. (**a**) The immunohistochemical staining of type I collagen in the myocardial infarction area. (**b**) The statistical analysis of type I collagen distribution in the myocardial infarction area. **** *p* < 0.0001. (**c**) The statistical analysis of type I collagen distribution in the border zone. **** *p* < 0.0001.

Fibrosis is a major pathological change observed during the end stage of MI, in which type I collagen assembles into dense and rigid fibres, causing collagen deposition. Through immunohistochemistry, we discovered that the distribution level of type I collagen in the infarcted area was 85.1 ± 7.6% in the PBS, 64.7 ± 6.0% in the RLP12 hydrogel, 66.7 ± 5.3% in the BADSC, and 50.9 ± 4.4% in the RLP12 hydrogel + BADSC groups (Figure 5a). The statistical analysis showed that compared to the PBS group, the distribution level of type I collagen at the MI site in the three groups was significantly reduced. The proportion of type I collagen in the RLP12 hydrogel + BADSC group was considerably lower than that in the RLP12 hydrogel and BADSC groups (Figure 5b). In the border area of MI, the distribution level of collagen I was 42.4 ± 5.8% in the PBS, 32.1 ± 4.5% in the RLP12 hydrogel, 30.9 ± 4.9% in the BADSC, and 21.1 ± 3.0% in the RLP12 hydrogel + BADSC groups. Further analysis showed that compared with that in the PBS group, the distribution level of type I collagen in the border area of the MI was significantly decreased in the other groups, and the type I collagen in the RLP12 hydrogel + BADSC group was significantly reduced compared to that in the other three groups (Figure 5c). The distribution of type I collagen aligned with the collagen deposition level, suggesting that either the RLP12 hydrogel or BADSCs, alone or in combination, can effectively mitigate fibrosis in the infarcted tissue.

### 2.6. Distribution Levels of Type III Collagen in the Infarct Area and Border Area

**Figure 6:**
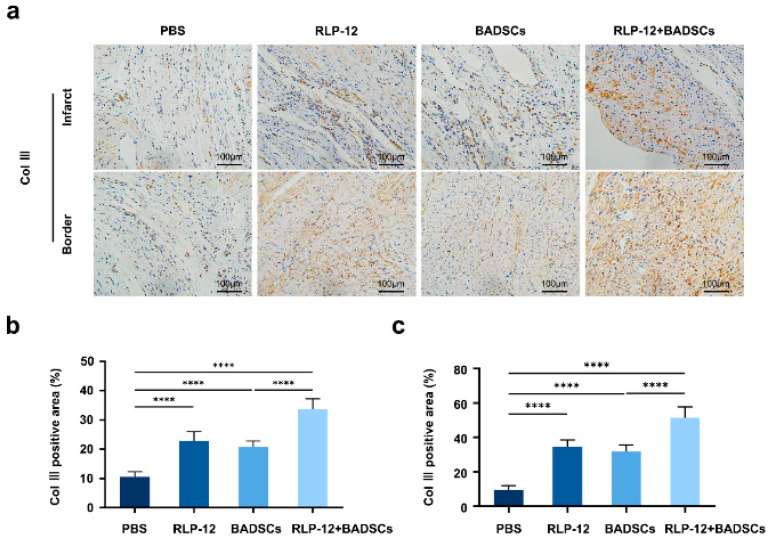
The distribution level of type III collagen in the myocardial infarction and border zone at 4 weeks after treatment. (**a**) The immunohistochemical staining of type III collagen in the myocardial infarction area. (**b**) The statistical analysis of type III collagen distribution in the myocardial infarction area. **** *p* < 0.0001. (**c**) The statistical analysis of type III collagen distribution in the border zone. **** *p* < 0.0001.

In contrast, type III collagen forms fine and pliable fibres that are crucial for maintaining tissue elasticity (Figure 6a). The collagen III distribution was 10.5 ± 1.8% in the PBS, 22.9 ± 3.1% in the RLP12 hydrogel, 20.7 ± 2.1% in the BADSC, and 33.7 ± 3.5% in the RLP12 hydrogel + BADSC groups. This indicates a significant increase in the distribution level of type III collagen at the site of MI after four weeks of treatment with the RLP12 hydrogel alone, BADSCs alone, and BADSCs carried by the RLP12 hydrogel when compared with that in the PBS group. Furthermore, the type III collagen distribution level was significantly higher in the RLP12 hydrogel + BADSC group than in the RLP12 hydrogel and BADSC groups (Figure 6b). In the border area of MI, the distribution level of type III collagen in each group was 9.5 ± 2.5% in the PBS, 34.5 ± 4.1% in the RLP12 hydrogel, 31.8 ± 3.8% in the BADSC, and 51.5 ± 6.3% in the RLP12 hydrogel + BADSC groups. The distribution level of type III collagen at the border area of the MI was significantly increased in the RLP12 hydrogel, BADSC, and RLP12 hydrogel + BADSC groups after four weeks of treatment. The distribution of type III collagen in the RLP12 hydrogel + BADSC group was significantly higher than that in the other three groups (Figure 6c). The distribution of type III collagen showed that its content could be increased by injecting the RLP12 hydrogel and BADSCs alone or in combination, which indicated that this strategy was helpful in maintaining myocardium compliance after MI.

### 2.7. BADSC Survival Rate and In Vivo Differentiation after Transplantation

**Figure 7:**
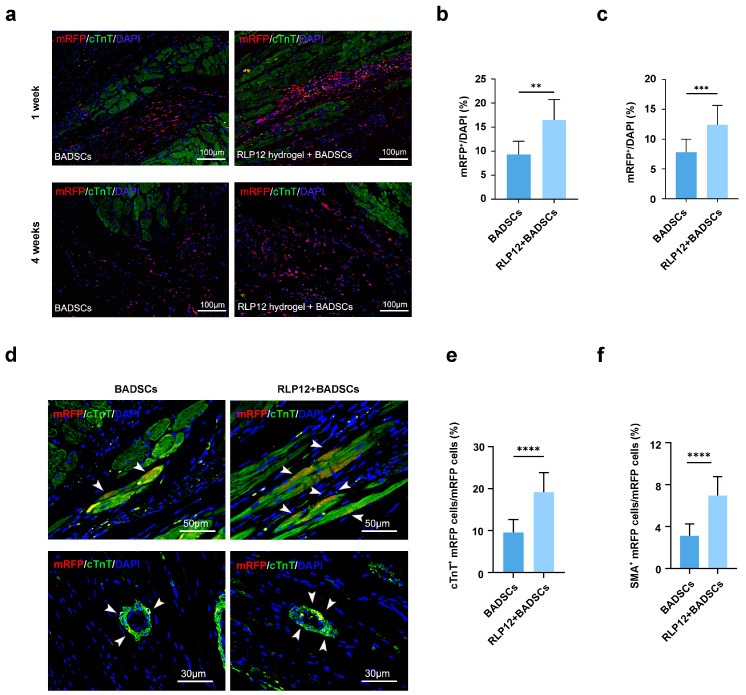
The cell survival and differentiation of BADSCs transplanted into infarcted myocardium. (**a**) Representative images of fluorescence microscopy at 1 and 4 weeks after the RLP12 hydrogel + BADSC treatment (scale bar = 100 µm). (**b**) The statistical analysis of the percentage of mRFP-positive cells to DAPI-stained cells at 1 week after treatment. ** *p* < 0.01. (**c**) The statistical analysis of the percentage of mRFP-positive cells to DAPI-stained cells at 4 weeks after treatment. *** *p* < 0.001. (**d**) Representative images of the immunofluorescence staining of cTnT-positive cardiomyocyte differentiation in the BADSC and RLP12 hydrogel + BADSC groups (myocardial infarction area, scale bar = 50 μm); representative immunofluorescence staining images of α-SMA-positive vascular smooth muscle cell differentiation in the BADSC and RLP12 hydrogel + BADSC groups (myocardial infarction area, scale bar = 30 μm). (**e**) The quantitative analysis of myocardial differentiation in the BADSC and RLP12 hydrogel + BADSC groups. **** *p* < 0.0001. (**f**) The quantitative analysis of vascular smooth muscle cell differentiation in the BADSC and RLP12 hydrogel + BADSC groups. **** *p* < 0.0001.

Representative images of surviving BADSCs are shown in Figure 7a. A week after transplantation, the mRFP-positive cells in the RLP12 hydrogel + BADSC group accounted for 16.5 ± 4.2% of DAPI-stained cells and mainly distributed in the myocardium. The mRFP-positive cells in the BADSC group accounted for 9.3 ± 2.7%, which were more dispersed than those in the RLP12 hydrogel + BADSC group (Figure 7b). After four weeks of transplantation, the RLP12 hydrogel + BADSC group exhibited a percentage of mRFP-positive cells in DAPI-stained cells at 12.4 ± 3.3%. This proportion is significantly higher than that of the BADSC group (7.8 ± 2.2%). In the RLP hydrogel + BADSC group, the BADSCs exhibited even dispersion in the infarct and border areas (Figure 7c). Conversely, in the group treated with BADSCs alone, the transplanted cells were primarily localised in the border area. These results indicate that the RLP hydrogel can lead to a substantial enhancement in the viability of BADSCs, both at one and four weeks after transplantation.

Four weeks after treatment, the specimens were collected, and the differentiation of BADSCs when injecting cells alone and alongside the RLP12 hydrogel in vivo was assessed through immunofluorescence staining (IF) (Figure 7d). The percentage of differentiated mRFP cells in the sections was calculated using Image Pro software (version 6.0). Our results showed that mRFP-positive and cTnT-positive cells matched in both the BADSC and RLP12 hydrogel + BADSC groups, indicating that BADSCs injected into the myocardium differentiated into cardiomyocytes (Figure 7e). The quantitative analysis showed that the number of cTnT-positive cardiomyocytes differentiated in the RLP12 hydrogel + BADSC group was significantly higher than that in the BADSC group (19.2 ± 4.7% vs. 9.5 ± 3.1%). In addition, the observed α-SMA-positive cells and mRFP-positive cells in the BADSC group and RLP12 hydrogel + BADSC groups matched, indicating that the BADSCs may differentiate into vascular smooth muscle cells (Figure 7f). Our quantitative analysis shows that the number of α-SA-positive BADSCs in the RLP12 hydrogel + BADSC group is significantly higher than that in the BADSC group (8.7 ± 2.3% vs. 3.9 ± 1.4%).

### 2.8. Microvessel Density (MVD)

**Figure 8:**
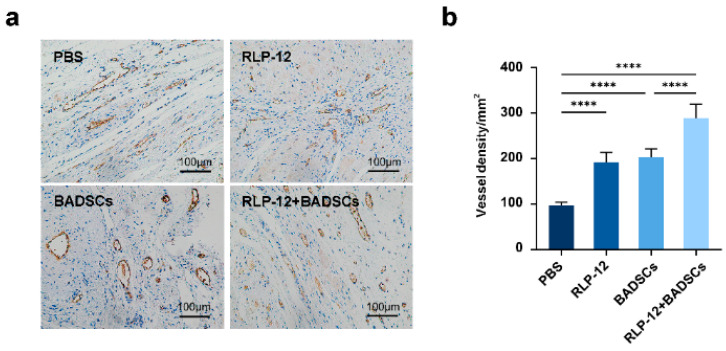
Vascularisation levels assessed using immunohistochemistry. (**a**) Representative images of MVD in the infarction area of MI rats after treatment. (**b**) The statistical analysis of the MVD in each group, ****p < 0.0001.

Capillaries are the smallest blood vessels, which are densely distributed in the cardiovascular system [41]. In order to evaluate the capillary status of an infarct heart after treatment, vWF immunochemistry staining was performed after 4 weeks of treatment (Figure 8a). The results show that the MVD at the infarct site was 96.3 ± 7.7/mm^2^ in the PBS group, 191.5 ± 22.0/mm^2^ in the RLP12 hydrogel group, 203.1 ± 18.3/mm^2^ in the BADSC group, and 288.9 ± 30.7/mm^2^ in the RLP12 hydrogel + BADSC group (Figure 8b). Statistical analyses showed that compared with that in the PBS group, the MVD was significantly increased in the other three groups. The MVD of the RLP12 hydrogel + BADSC group was significantly higher than that of the other three groups.

In this study, we prepared an RLP12 hydrogel based on resilin for injectable myocardial tissue engineering. First, an RLP12 protein expression vector was constructed, and the RLP12 protein was obtained after induction and purification. The molecular weight of the RLP12 protein was between 25 and 34 kDa, consistent with the reported 27.5 kDa [42].

THP was used as a crosslinking agent in the formation of an elastin-like polypeptide gel. It was discovered that the gel could adequately maintain the viability of mouse fibroblast cultures in vitro, indicating that THP has low cytotoxicity [43]. Moreover, the subcutaneous injection of the RLP12 hydrogel obtained by THP crosslinked into mice did not cause noticeable inflammatory responses, proving its good biocompatibility [36]. Importantly, the mechanical strength (10–15 kPa) of the *RLP12 hydrogel is consistent with the myocardial tissue (7.8 ± 4.1 kPa -26.2 ± 5.1 kPa) of rats in the state of natural pulsation [44,45]. Therefore, we selected the RLP12 hydrogel for further studies.

Studies have confirmed that MI forms a microenvironment of local ischemia, hypoxia, and extracellular matrix degradation [46]. Within this context, it is difficult for stem cells to survive and function when injected by themselves. Therefore, myocardial tissue engineering using hydrogels carrying stem cells for in situ injections has received increasing attention [47,48]. However, dealing with fibrosis and the local microenvironment following MI remains challenging. Uncontrollable negative ventricular remodelling after MI is the pathological basis for progression to heart failure. Both biological and mechanical malignant microenvironments are involved in negative ventricular remodelling [47]. Currently, most injectable materials are designed to protect biological microenvironments. However, a mechanical microenvironment is typically formed, which promotes the development of heart failure. Recently, studies have discovered that elastic hydrogels can also provide mechanical assistance for infarct tissues owing to their elasticity and extensibility. This was associated with improved local myocardial dyskinesia and reduced stress on the ventricular wall, which is conducive to the improvement of cardiac function [49]. A study on the inter-impact of the hydrogel and the stress and thickness of the infarcted ventricular wall showed that a hydrogel with a stiffness of less than 50 kPa can significantly reduce the stress on muscle fibres [24]. In 2019, Matsumura et al. injected a synthetic elastic hydrogel material poly(NIPAAm-co-HEMA-co-MAPLA) into the MI site of a miniature pig MI model and discovered that it could improve the mechanical strength of the infarcted tissue and reduce the formation of scar tissue, improving ventricular remodelling and cardiac function [50]. In addition, the mechanical signals of viscoelastic hydrogel could regulate the directed differentiation of tissues [51]. In this study, we used an RLP12 hydrogel as an injectable vehicle of BADSCs to the MI site of rats. We observed that the RLP12 hydrogel alone or with BADSCs significantly decreased the fibrosis level in the infarct area and improved cardiac function, which may be related to the RLP12 hydrogel in the mechanical microenvironment of the infarct tissue. Studies have shown that resilin hydrogels have a significant repair effect on elastic tissues. In 2019, King et al. combined a resilin-like protein with hyaluronic acid (HA) to produce RLP/HA and RLP-acrylamide/mercaptanisation HA (RLP-AM/HA-SH) composite hydrogels with elastic shear moduli of ~600 and ~1500 Pa, respectively. These hydrogels have a similar elastic shear to that of human vocal cord tissue (400–2000 Pa). The hydrogels could maintain the viscoelasticity of the vocal cord when being injected into vocal cord tissue [52]. The shear modulus of the RLP12 hydrogel used in this study was 10–15 kPa, which is similar to the elastic modulus of rat myocardium in the state of natural pulsation, which ranges between 7.8 ± 4.1 and 26.2 ± 5.1 kPa. Therefore, the RLP12 hydrogel can satisfy the mechanical compliance requirements of the myocardial tissue.

The excessive deposition of denatured collagen leads to fibrosis, with type I collagen mainly deposited, whereas type III collagen is beneficial for maintaining tissue elasticity [53]. The ratio of Col I to Col III in the healthy myocardium is lower than that in the infarct area of the myocardium [54]. Research has shown that a high Col III/I ratio is commonly observed in young rats, and high expression of type III collagen is beneficial for reducing fibrosis [55]. Wen et al. suggested that an elastic Dex PCL-HEMA/PNIPAAm (DPHP) hydrogel could improve the distribution of type III collagen after transplantation to the infarct site [56]. In this study, the distribution of type I and III collagen in the heart slices of each group was analysed. The results show that the content of type III collagen in the infarct site and the border area increased, indicating that it can maintain the compliance of myocardial tissue after MI, further confirming that the RLP12 hydrogel improved the mechanical microenvironment. Although the content of type III collagen increases and can alleviate fibrosis to some extent, excessive deposition of type III collagen may cause myocardial hypertrophy, leading to cardiac dilation and heart failure [57].

We believe that RLP12 hydrogels, as injectable vehicles, can significantly improve the cardiac function of MI rats, primarily in the following aspects. First, the RLP12 hydrogel can form a gel in situ, improving the survival of BADSCs and promoting the role of BADSCs in myocardial repair. Second, the RLP12 hydrogel can alleviate fibrosis by improving the mechanical microenvironment of the MI and the compliance of the myocardium by increasing the distribution of type III collagen. Third, improving the mechanical microenvironment can optimise the biological microenvironment, thereby promoting the differentiation of BADSCs into cardiovascular lineage cells and increasing the MVD in the infarcted area. Next, in order to develop the functional materials based on the resilin hydrogel with superior physical/chemical properties and biological functions, we will carry out the studies related to the properties and degradation of RLP hydrogels with different protein concentrations and crosslinking degrees, the interaction between hydrogels and stem cells, and evaluate their function on repairing damaged myocardium in large animal MI models.

## 3. Conclusion

In this study, the RLP12 hydrogel combined with BADSCs significantly improved cardiac function by increasing microvessel density and reducing infarct area size and collagen deposition in MI rats four weeks after transplantation. The distribution ratio of collagen III to I increased in both the centre and edge areas of the MI, indicating improved compliance of the infarct heart. This study provides novel insights into the mechanical interactions between the hydrogel and the infarct microenvironment.

## 4. Materials and Methods

### 4.1. Expression and Purification of RLP12 Protein

The RLP12 protein sequence reported by Charati et al. was selected [41], with a total of 822 bp. BamH I and Hind III cleavage sites were added at both ends, synthesised by the GenScirpt Company (Nanjing, China), and inserted into the pUC57 cloning vector (Figure 1). Double enzyme digestion of the pET-28a vector and RLP12-pUC57 was performed to recover the target fragments. The T4 DNA ligase was used to link the pET-28a vector fragment (carrying 6xHis tag) with the RLP12 fragment, and the sample was incubated overnight at 16 °C. The recombinant expression vector was transfected into *Escherichia coli* BL21 (DE3) cells. The bacterial solution obtained was subjected to nucleic acid sequence analysis. The positive clone was identified as *E. coli* BL21 (DE3)-RLP12-pET-28a. The plasmid was extracted, and double restriction enzyme identification was performed. To analyse and compare existing methods for expressing the resilin protein, we selected the automatic induction method and referred to a previous study that introduced the use of the ZYP-5052 automatic induction medium (Sinopharm Chemical Reagent Co., Ltd., Shanghai, China) to express the RLP12 protein [36]. RLP12 protein was extensively expressed and extracted. The target protein was purified using a Ni^+^ column (Qiagen, Germany), and SDS-PAGE was performed.

### 4.2. Preparation and Identification of the RLP12 Hydrogel

First, we desalinated and concentrated the RLP12 protein. We selected a centrifugal filter (Amicon^®^ Ultra-15 10 K, Millipore, Burlington, MA, USA), added the protein sample to the filter (≤15 mL), and centrifuged the sample at 4 °C at 4000× *g* for 15–40 min. Deionised water was added to the concentrated solution up to the original volume of the sample, and this was repeated several times until the salt content decreased. Concentrated protein samples (150 μL to 1 mL) were freeze-dried overnight. The protein content in each tube was measured. The RLP12 hydrogel was prepared by dissolving lyophilised RLP12 protein in PBS, adjusting the concentration to 100 mg/mL, and preparing THP and PEG solutions. Before mixing, each solution was placed on ice to reduce the reaction rate and prevent excessive crosslinking. A 10 wt% hydrogel was prepared by vortexing the ingredients. Subsequently, the hydrogel was incubated at 37 °C for crosslinking for 1 h.

### 4.3. Examination of Mechanical and Rheometric Properties

The RLP12 hydrogel was analysed using a rheometer (Thermo Fisher, Waltham, MA, USA). At 37 °C, dynamic oscillation time, frequency, and strain scanning were assessed using a C35/1° cone-plate insulation sleeve. The total volume of each sample was 200 μL. The mixture was transferred to the bottom plate of the rheometer for in situ rheological analysis. Strain scanning measurements were performed on each sample from 0.01% strain to a maximum of 1000% strain to determine the linear viscoelastic region (LVE). Time scanning at a fixed strain amplitude of 1% in LVE (t: 0–8000 s, ω: 6.28 rad/s) and frequency scanning experiment (ω: 0.1–100 rad/s) were performed to assess the rheological properties of the hydrogel. The experiments were repeated thrice for each sample.

### 4.4. Establishment of a Rat MI Model and Injection

All animal experiments were approved by the Animal Ethics Committee of the College of Life Sciences and Bioengineering (approval number SS-HBW-2018-07). A rat model of MI was established as previously reported [58]. Rats were anaesthetised for surgery; the ligation was performed using a 6-0 Prolene suture (Lingqiao, Zhejiang, China) between the rat pulmonary artery cone and the left atrial appendage at 2–3 mm from the beginning of the coronary artery to 2 mm below the left atrial appendage and at a depth of 0.5–1 mm. The colour of the left ventricular anterior wall myocardium changed to cyan, followed by whitening, indicating success. MI rats were randomly divided into four groups: (1) control (PBS, *n* = 12); (2) RLP12 hydrogel (*n* = 12); (3) BADSCs (*n* = 18); and (4) RLP12 hydrogel +BADSCs (*n* = 18). Six animals from groups 3 and 4 were collected one week after surgery to evaluate the survival rate of the transplanted cells. The remaining rats underwent cardiac ultrasound examination four weeks after surgery, followed by sampling for histological and immunohistochemical testing.

### 4.5. Cardiac Function Testing

After four weeks of postoperative feeding, the cardiac function of the rats was tested using the Vevo 1100 ultrasound imaging system (VisualSonics, Toronto, Canada). After anaesthesia, the skin of the rats was prepared, and an appropriate amount of ultrasound gel was applied. When the heart rate stabilised, ultrasound images were collected, and the electrocardiogram and heart rate were monitored. Guided by two-dimensional images, M-shaped curves were measured to determine the left ventricular end-systolic diameter (LVESD), left ventricular end-diastolic diameter (LVEDD), left ventricular fractional shortening (LVFS), and left ventricular ejection fraction (LVEF). Subsequently, 3–6 cardiac cycle measurement data points were collected from each rat, and the average value was calculated. The formulas were as follows: LVFS (%) = [(EDD − ESD)/ EDD] × 100 and LVEF (%) = [(LVIDd)^3^ − (LVIDs)^3^]/(LVIDd)^3^ × 100.

EDD: End-Diastolic Diameter;
ESD: End-Systolic Dimension;
LVIDd: Left Ventricular Internal Diameter in Diastole;
LVIDs: Left Ventricular Internal Diameter in Systole.

### 4.6. Histological Testing

After the cardiac function assessment, rats were euthanised using a 2% potassium chloride solution, and the heart was collected. The ventricular wall below the trisection ligation, along the short axis of the heart, was immersed in 4% paraformaldehyde. Samples were cleaned, dehydrated with distilled water, and sliced in paraffin. Subsequently, Masson’s trichrome staining was performed, and slice images were acquired. The fibrosis level and infarcted area were determined using Image Pro analysis software (version 6.0). The formula used is as follows: myocardial infarction size = (left ventricular endocardial length in the infarcted area/left ventricular intact endocardial circumference) × 100. Collagen deposition was evaluated using Image Pro software. We calculated the percentage of the left ventricular fibrosis area in the left ventricle area. Five cross-sections were selected from the bottom to the apex of each heart specimen for immunohistochemical staining of type I and III collagen. After image acquisition, we calculated the percentage of positive areas of the two types of collagens in the left ventricular MI area and the MI edge area using Image Pro analysis software. Using the same method for vWF immunohistochemistry staining, the number of microvessels with a diameter of 10–100 µm in the MI area was assessed, and the MVD of each group was calculated.

IF staining was performed using cardiac troponin T (cTnT) antibodies. The percentage of red fluorescent cells among the DAPI-stained cells was calculated using Image Pro analysis software. Using this information, we evaluated the survival of BADSCs at different time points. IF staining with α-SA antibodies was performed to detect the differentiation of BADSCs in vivo. We calculated the number of mRFP-positive myocardial cells (cTnT + mRFP) and the number of mRFP-positive vascular smooth muscle cells (α-SA + mRFP).

### 4.7. Statistical Analysis

The data were expressed as mean ± standard deviation and analysed using the SPSS software (version 17.0, IBM, Armonk, NY, USA). Statistical differences between two groups were analysed using a *t*-test; comparisons between two or more groups were analysed using a one-way ANOVA combined with the least significant difference (LSD) test. Values of *p* < 0.05 were considered significant.

## Data Availability

The original contributions presented in the study are included in the article, further inquiries can be directed to the corresponding authors.
